# Book and Pamphlet Notices

**Published:** 1860-05

**Authors:** 


### BOOK AND PAMPHLET NOTICES.

Clinical Lectures on certain Acute Diseases; by Robert Bently Todd, M. D., F. R. S., Author of Lectures on Diseases of the Urinary Organs, etc., formerly Physician to King's College Hospital, London. Blanchard & Lea, Philadelphia, 1860. Received through S. C. Griggs & Co., 39 & 41 Lake St.

This book contains a series of Dr. Todd's Clinical Lectures, delivered in King's College Hospital, on the following subjects:

Rheumatic Fever, Continued Fever, Erysipelas, Acute Inflamation, Pyæmia, Pneumonia and its complications, and the Therapeutical action of Alcohol.

The presentation of this work to the professional public, is a fitting close to a life of honor and usefulness, of which we have so conspicuous an exomple, in the career of Dr. Todd. Though a less labored work than are most of those that have previously come from his pen, which, from their merit, have made his name so familiar to the world, yet it will hardly prove less useful; containing, as it does, the perfected knowledge on the subjects on which it treats, of an acute observer, endowed with industry and favored with every desirable opportunity for arriving at truthful conclusions.

The diseases considered are among the most isteresting with which it is the fortune of every physician in active practice, to come in daily contact. The treatment adopted in the different diseases, and its results are given; the physiology of organs is indicated, and the pathology of diseases illustrated by numerous clearly reported cases.

After giving a clinical history of Rheumatic Fever, the different modes of treatment that have been most resorted to in its management, are reviewed:

1st. Extreme depletion by venesection. This is deemed pernicious, being productive of harm rather than good.

2d. Moderate bleeding and the administration of diaphonetics. So far as the bleeding goes, this is thought to be injurious, and the diaphonetics prove useful only to the extent they may may promote elimination. On the whole, it is an expectant treatment and is somewhat better than the first.

3d. By mercurials, to which it is objected "that it is an attempt to cure one fever by setting up another, and in some respects, a worse. Even supposing the original disease succombs, your patient comes out of his rheumatic fever with loose teeth, ulcerated gums, and all the painful concomiants of ptyalism. Now I say, that under these circumstances, the remedy is nearly as bad as the disease; and, moreover, it does not in the least guard the patient against what may be termed the accidents of his malady—those severe internal inflamations, pericarditis, endocarditis, pneumonia, pleusitis, peritonitis."

4th. By colchicum and guaiacum—these are inefficient, unless carried to an extent to occasion purging, when they do good as eliminitives, but they sometimes induce a dangerous degree of prostration.

5th. Opium—an excellent remedy, but it should not be relied upon alone.

6th. Quinine—not so universally useful as opium, but in many cases an agent ot great worth.

At the conclusion of such a review, can we wonder that the exclamation should be made, "imagine the state in which the pithology of a disease must be, when measures so completely at the opposite extremity of our therapeutical resources are advocated for it, as venesection to the amount of two or three parts on the one hand, and large doses of quinine on the other." The treatment that Dr. Todd believes to be the most beneficial, is that by *elimination*; its adoption being based upon the idea that the disease is due to some "*materies morbi*" in the blood, "probably lactic acid, or some anologous agent." This is saught to be removed by the "promotion of the action of the skin, the kidneys, and the bowels, and the use of antacid remedies, and large quantities of fluid for the free dilution of the materies morbi, and to supply the waste caused by the drainage from diaphoresis and diuresis." In the other diseases, especially typhoid fever and erysipelas, Dr. Todd recommends the early adoption of a vigorously sustaining treatment.

The views of the pathology of these diseases, and the treatment proper for them, cannot be said to be new, for recently the general practice of the profession has been verging more and more to the general treatment; and this we deem a vast improvement on the antiphlogistic plan formerly so univerresorted to, and even yet, not sufficiently discarded. Dr. Todd may go farther than many practitioners will be wllling to follow in the application of this plan of treatment; but this we believe, that in the main, he is correct; and that more especially in typhoid fever and erysipelas great numbers of patients are annually sacrificed by a failure to recognize the necessity of an early, vigororus and persistent support of the vital powers.

Dr. Todd's views of the physiological action of alcohol ore embraced in the following quotation, which indicates his idea of its therapeutical application; page 290:

Alcohol, taken carefully, incseases the animal temperature; it also strengthens the action of the heart; and, when administered under proper circumstances, it reduces the frequency of the pulse. So far as it influences the nervous system, the action of alcohol is that of what is commonly called a *stimulant*, an unfortunate term, indicating a distinction without a difference. Other forms of food are likewise stimulant, but as they do not act directly and quickly on the nervous system, their exciting properties are not so apparent. In like manner, alcohol possesses its stimulating property because it is a form of aliment, appropriate to the direct nourishment of the nervous system, and to its preservation; and its special adaption to this system gives it an immediate exciting power superior to any other kind of food. Alcohol is also fitted to uphold the calorifacient process, and therefore to protect the nervous and other tissues, which, without it, would be largely called upon to furnish fuel for the oxygen which supports that process. And for this purpose, alcohol possesses considerable advantages over most other hydrocarbonaceous substances, in consequence of the ready manner in which, when swallowed, it passes into the blood. Oil is a hydrocarbon equally with alcohol; but the digestion of the latter is a very different affair from that of the former. Oil, when swalloed, remains for some time in the stomach, where, however, it undergoes no change; it subsequently passes down into the small intestine, where it is subjected to the action of the pancreatic and other fluids, and having become converted into chyle, is absorbed by the intestinal villi. Thus the digession of oil is a far more complicated porcess than that of aicohol. Hence, then, as a calorifacient form of food, as a promoter of the nutrition of the nervous system, and as admitting of easy and quick absorption into the blood, alcohol possesses a combination of qualities which render it of the utmost value in tne treatment of disease.

The subjoined is the substance of the treatment recommended for erysipelas; page 142:

The upshot, then, of all I have to tell with respect to the treatment of erysipelas, is to give stimulants and nourishing freely, and from the very commencement of the attack.— Don't trouble yourselves with too much attention to the secretions, as some are apt to do, who imagine that the alteration of these by gray powder, black draught, *et hoc genus omne*, is necessary to the favorable issue of the case, but who, by the time they have got the secretions into what they conceive to be a correct condition, find that their patient is fairly slipping through their fingers, and is dying, worn out and exhausted. As soon as you are satisfied that the patient to whom you are called is laboring under erysipelas, at once begin to administer stimulants and nourishing food, using the precautions I have mentioned; and what I wish above all things to impress upon you is, that this stimulating treatment should be employed from the very beginning of the attack. With respect to the bowels, you must be guided by circumstances; if they are confined, you may open them by an enema, or by a dose of castor oil or some other medicine, which will neither irritate the mucous membrane of the alimentary canal, nor exhaust the patient's strength; always keeping in view that the poison of erysipelas is exceedingly depressing in its action, and that the object of all your treatment should be, first, to antagonize the poison, and seconly, to uphold the patient's powers, to enable him to bear up against one of the most lowering and debilitating diseases to which the human frame is liable.

Now of all the stimulants, I believe, as I have already said, the alcoholic are the best, and I have witnessed such remarkable effects, in such a variety of cases, produced by their free exhibition, that I am inclined to consider them as *antidotes* to the erysipelatous poison. If I were to be restricted to any one remedy in the treatment of this disease, I should, assuredly, use brandy. With a commissariat well supplied with brandy, and simple means to keep the bowels open, I think I could engage to keep erysipelas at a minimum among the wounded in our army in the Crimea.

Some attach great importance to the use of the tincture of sesquichloride of iron in this disease. I have no doubt many cases, such as those which I have placed in my first group, will get well under that drug, partly and mainly because it excludes depressing treatment, partly, perhaps, from some tonic power in the medicine; but I would as soon think of trusting to it in the third or fourth group of cases, as I would to the billionth of a grain of anchorite, or arnica, or sulphur, or any other homœpathic absurdity. The remedy, so far as I know, is unobjectionable in itself, but its power to do good is small; and if you try it, let me advise you not to trust to it alone, but merely to use it as an adjunct to the treatment which I have endeavored to impress upon you to-day. For, as I before said, there is a large class of cases of erysipelas which will get well without any treatment whatever, and, indeed, in spite of the depressing treatment, either because the dose of the poison which these patients have imbibed has been very small, or because their powers of resisting acute diseases are very great. In such cases, you may, if you like, amuse yourselves with giving a remedy of the nature of sesquichloride of iron. But in all severe examples of the malady, place your trust in food and brandy, freely given under careful regulation, and adopted from the commencement of the attack.

A GUIDE TO THE PRACTICAL STUDY OF DISEASES OF THE EYE—WITH AN OUTLINE OF THEIR MEDICAL AND OPERATIVE TREATMENT. BY JAMES DIXON, F. R. C. S., SURGEON TO THE ROYAL LONDON OPTHALMIC HOSPITAL, MOORFIELD. FROM THE SECOND LONDON EDITION. PHILADELPHIA; LINDSAY & BLAKISTONE, 1860. FROM S. C. GRIGGS & CO., 39 AND 41 LAKE ST.

Few diseases have so much interested the medical profession of England and the continent of Europe, within the past few years, and been the subject of works of merit, as those of the eye. When we reflect that there is scarcely a branch in the whole range of medical science of so much practical importance as opthalmology, that there is scarcely a nonvital organ in the body, upon which the greater portion of mankind is so dependant as upon the eye, not only for moral and mental culture, but for daily support, it seems strange that the study of this class of diseases should have been so neglected as they have been by the profession of this country. Physicians in every part of the country acknowledge that too little interest is felt in this subject. It can be explained in part, we think, by this neglect of ophthelmic science, why the treatment of diseases of the eye falls so extensively into the hands of a class of practitioners, which is not regarded as honorable by the profession.

Fortunately, a greater interest in the subject is awakened in different parts of the United States. Infirmaries have been established in several eastern cities, affording excellent facilities for clinical instruction, the importance of which is also impressed upon the mind of the student.

Every new work upon diseases of the eye, especially adapted to the wants of the student must be received with favor by the friends of medical science. We believe that text books with the leading principles of each department of medicine, carefully explained, are of the greatest practical utility to young men while pursuing their college course.

As regards the large standard works upon diseases of the eye, it is undoubtedly true that the student, while attending lectures, has not time to peruse any one of them with care.

The book before us we consider as being particulaply fitted to fill the place for which it was intended. It is, as it purports to be, a guide to the practical study of diseases of the eye. The volume, very much smaller than ordinary medical works, equals, perhaps, in typography and general appearance, any medical publication we have.

The work gives a concise statement of nearly all the great discoveries in the surgical treatment of ophthalmic disease within the past few years. The principlns and use of the ophthalmoscope are clearly, though briefly explained, and its utility in diagnosis fully appreciated.

The volume is of such a size, and the principles which it teaches are so clearly defined, that the student can reasonably be expected to acquire a thorough knowledge of all it contains, before finishing his college course.

The diseases of no organ demand more careful clinical observation on the part of the student in acquiring skill in diagnosis, than those of the eye. It is only in connection with suitable clinical instruction that the student can gain such practical knowledge from any work on diseases of the eye, as to enable him to diagnose these diseases with accuracy and treat them with judgment.

We do not feel called upon to criticise the work on account of many omissions which we might notice, since the author claims for it no higher title than that of *Guide*, with an *outline* of medical and surgical treatment.

While we would recommend the work of Mr. Dixon to the *student*, we would say to the physician that it should not take the place of Graefi, Sichel Arlt, Dresmarres, and the reprints of English writers. It is trom the careful study of such works alone, that a thorough knowledge of the physiology, palthological anatomy and treatment of diseases of the eye can be learned.

E. L. H.

A Treatise on Medical Electricity, Theoretical and Practical; and its uses in the treatment of Paralasis, Neuralgia, and other diseases. By J. Althaus, M. D., Philadelphia. Lindsay & Blakiston. From S. C. Griggs, 39 and 41 Lake Street.

It is the province of the intelligent physician to apply all substances and forces in the whole range of nature to the prevention, mitigation, or cure of disease; and herein lies the difference that exists between the medical profession and the numerous fungous excresences that have in all times protruded, one after another, upon its surface, existed for a brief time, and sloughed away, as those now disfiguring it are, in their turn, destined to do. It is not strange that an agent of such wondrous powers as electricity, should, soon after it began to arrest attention, hove been applied as a relief to disease and suffering, yet it is something so subtle in its nature as not easily to be investigated by the mass of observers; its properties and powers being known only by the results of its action. Therefore it is not strange that its uses as a remedial agent have hitherto been chiefly of an empirical, and therefore, not very intelligent character.

The work under notice is evidently the offspring of much thought on the subject, and will do something to improve our knowledge of the circumstances demanding the use of this remedy, and the proper mode in which it should be applied. We may also reasonably expect that some good may be accomplished by the tendency it will have to direct the attention of the profession more especially in this channel.

Dr. Brown's Sequard's Journal of Physiology.

We have received the first No. of this publication, for the year 1860. It continues to sustain its high character. We are pleased to learn that it has been so successful as to give assurance of permanency, and to enable the proprietor to add to its value by more copious and expensive illustrations.

The chief editor has been appointed physician to the hospital for epileptics, in London, and has recently removed his residence to that city; but this will nor affect the Journal, which will be issued, as before, at Paris.

